# Socialising kitties: A quantitative survey of US cat owner attitudes towards kitten and adult cat socialisation programmes

**DOI:** 10.1017/awf.2025.10013

**Published:** 2025-06-23

**Authors:** Jennifer K Link, Carly M Moody

**Affiliations:** The Animal Welfare Epidemiology Laboratory, Department of Animal Science, University of California, Davis, CA, USA

**Keywords:** Animal welfare, behaviour, cat management, critical period, feline, human-animal interactions, sensitive period

## Abstract

Socialisation is important for normal social and behavioural development in companion animals. However, little research has focused on kitten socialisation or owner attitudes towards kitten socialisation programmes. Thus, we used a quantitative online survey to describe US cat owner attitudes towards kitten socialisation and elucidate aspects of socialisation programmes deemed important by owners. Questions (n = 45) included participant demographics, participant experiences with socialisation, information regarding participants’ cats (*Felis catus*), where participants receive socialisation information, and rating the importance of socialisation components. Participants were recruited via advertisements posted on social media and an online news article. Of the 2,238 responses, participants were most frequently women (74.7%), owning two cats (38.0%), who had not worked with cats professionally (72.9%). Most participants had not heard of socialisation programmes for kittens (69.3%), but would be interested in enrolling in a future programme (50.4%). Participants indicated important aspects of kitten socialisation programmes as: education about reducing problem behaviours (87%); understanding cat body language (85.8%); and getting kittens used to handling (83.1%). A logistic regression revealed that the presence of aggression in their current cat predicted interest in a future programme, as well as living in an urban or suburban area. These results suggest a lack of owner awareness of kitten socialisation, and indicate many owners are interested in enrolling in a future kitten socialisation programme. Further research should explore methods to improve access to kitten socialisation information, elucidate components of current socialisation programmes, and assess their impact on owner management and cat behaviour.

## Introduction

Socialisation programmes are recommended by pet professionals and veterinary experts to help promote normal kitten social and behavioural development (Crowell-Davis 2007; Seksel 2008; Seksel & Dale 2012; Landsberg *et al.* 2013; American Veterinary Medical Association [AVMA] 2015). Socialisation refers to a learning process in which an animal develops valanced associations with people, animals, environments, and other stimuli. Socialisation is often discussed in the context of the sensitive period of socialisation (Karsh & Turner 1988; McCune 1995; Landsberg *et al.* 2013). A sensitive period refers to a specific time-frame in which the effect of experience on the brain is particularly strong. When experience during this time-frame is essential for normal development, the time-frame is described as a *critical* period (Knudsen 2004). This period is often characterised by an increase in neural plasticity (Rohlfs Domínguez 2014), with a gradual decline in plasticity into adulthood. Increased neural plasticity is characterised by an increase in the ability of the nervous system to be modified by experiences (von Bernhardi *et al.* 2017). When discussing the sensitive period of socialisation, most refer to a time-frame in which the effects of socialisation are particularly impactful. Some differences exist in the literature regarding whether the socialisation window is referred to as a sensitive (e.g. Foerder & Howard 2024; Graham *et al.* 2024a) or critical (e.g. Karsh & Turner 1988; Ahola *et al.* 2017) period. For the current study, this time-frame will be referred to as a sensitive period.

The proposed sensitive period of kitten socialisation is between 2–7 weeks old (Karsh & Turner 1988), or up to 9 weeks old (McCune 1995; AVMA 2024) and is sometimes described as being lifelong (Petak 2022; AVMA 2024), which implies the possibility of continuation past this window. Sensitive periods are characterised as functional descriptions of neurological mechanisms rather than specific age ranges and may be mediated via external environmental and biological factors (Hensch 2018; Gabard-Durnam & McLaughlin 2020). Thus, the end of the socialisation sensitive period is likely not static and may vary across individuals. Much of a kitten’s proposed socialisation sensitive period occurs before they are adopted or enter their forever home. Thus, socialisation experiences prior to entering a forever home are very important (Campbell *et al.* 2024), however new kitten owners may not receive information on these experiences. Kitten socialisation programmes are offered at eight weeks of age (Landsberg *et al.* 2013) and some programmes may accept kittens up to eight months old (Vitale 2022). Typically, classes like these are not offered for kittens younger than eight weeks old, likely due to the increased risk of disease (Landsberg *et al.* 2013). Since kittens are enrolled in socialisation programmes after the sensitive period of socialisation, experiences after this period may have reduced effectiveness compared to experiences during the sensitive period; however, no research has assessed this. In addition, many shelters do not allow adoptions of kittens younger than eight weeks of age, and many US states have legislation that prohibit the sale of kittens younger than eight weeks of age (e.g. Illinois, 225 I.L.C.S. § 605/2.2; Michigan, M.C.L. 287.335a; Minnesota, M. S. A. § 347.59).

Socialisation programmes provide an opportunity for kittens to develop positive associations with new stimuli, such as unfamiliar people, conspecifics, environments, and objects. Although the content of kitten socialisation programmes varies, kittens and owners often engage in a multi-week programme where owners learn about cat (*Felis catus*) health and behavioural management, and kittens are exposed to various stimuli, including a carrier, travel, an unfamiliar environment, unfamiliar people, and unfamiliar conspecifics (Seksel 2008; Seksel & Dale 2012; Landsberg *et al.* 2013). There is a lack of research examining the impacts of socialisation programmes on future kitten health and behaviour, although some research has examined the impact of various aspects of socialisation programmes on kittens and adult cats of varying ages. For example, research suggests that carrier training in cats older than eight months may reduce future stress during exposure to carriers and travel (Pratsch *et al.* 2018); kittens exposed to unfamiliar people show reduced fear responses to unfamiliar people later in life (Collard 1967); and kittens exposed to complex (versus barren) environments may show increased cognitive skills by greater success at a maze task (Wilson *et al.* 1965). Additionally, most behavioural research examining kitten socialisation has focused on exposures during the socialisation period, in particular, handling (Wilson *et al.* 1965; Karsh 1983; Karsh & Turner 1988; McCune 1995; Casey & Bradshaw 2008). This handling research suggests that handling kittens for 5–15 min per day during early life is associated with a reduced latency to approach novel humans (Wilson *et al.* 1965; Karsh 1983; Karsh & Turner 1988; McCune 1995).

Overall, socialisation programmes often prioritise owner education, which may be particularly beneficial for educating owners about cat behaviour, at-home management, and routine procedures (e.g. nail trims) needed throughout a cat’s life (Landsberg *et al.* 2013; Blackwell 2022). While many kitten socialisation programmes offer in-person classes, some organisations and behaviour professionals also conduct synchronous or asynchronous virtual classes. Virtual classes focus on owner education (i.e. litter-box maintenance, importance of vertical space, etc), provide guidance on socialisation activities to perform with kittens, and answer owner questions on kitten ownership and management. However, research is needed to elucidate the benefits and limitations of in-person versus virtual programmes on owner education and kitten socialisation experiences.

Although many socialisation programmes focus on kittenhood, some veterinary resources recommend continuing socialisation practices until at least 12 months of age (AVMA 2024). Limited research exists on the impacts of socialisation practices on neural plasticity into adulthood. One study in rats (*Rattus norvegicus*) suggests that housing with environmental enrichment versus standard laboratory caging in adulthood enabled an eye previously damaged due to sight deprivation during the sensitive period, to make a full recovery (Sale *et al.* 2007). This finding suggests that exposure to environmental enrichment into adulthood, a practice often included in the curriculum of socialisation classes, may continue to impact animal development past sensitive periods. However, given the lack of information on the impact of socialisation practices past kittenhood, there is a need for further research.

Although kitten socialisation is often recommended by pet care professionals, to date there has yet to be any research exploring cat owner attitudes towards socialisation programmes for kittens and cats. Thus, here, we surveyed US cat owners using an online quantitative questionnaire to examine: (1) owner experiences with kitten and cat socialisation programmes; (2) where owners receive their socialisation information; (3) interest in enrolling in a future socialisation programme and factors impacting interest; and (4) attitudes regarding important components of socialisation programmes.

We predicted that participant demographic factors would be most predictive of interest regarding a future kitten socialisation programme. We predicted that women would be more likely than men to indicate interest, given previous research has found higher support for animal welfare by women compared to men, not to mention the formation of greater attachments to animals (Eldridge & Gluck 1996; Knight *et al.* 2004; Herzog 2007). We also predicted that those who participated in a socialisation class in the past would be interested in doing so again in the future. Furthermore, we predicted that those in a higher socio-economic status would be more likely to indicate interest in a kitten socialisation programme due to an increased amount of disposable income. Similarly, those willing to pay a higher amount to participate in a kitten socialisation programme would be more interested in enrolling. Finally, we predicted that individuals whose current cat is experiencing behaviour problems would be more interested in enrolling in a future socialisation class as they may be seeking help with their cat’s behaviour.

## Materials and methods

This research was reviewed and deemed exempt (e.g. does not require registration) by the UC Davis Institutional Review Board Administration (IRB# 1883274). Participation was voluntary and respondents could withdraw at any time. Prior to completing the questionnaire, participants were presented with a consent form detailing the duration of the study and an overview of the types of questions they would be asked. Participants could only access the questionnaire if consent to participate was provided.

### Questionnaire

A quantitative questionnaire was created using online survey software (Qualtrics, Provo, UT, USA), and participants needed to be current cat owners living in the US who were 18 years of age or older. Questions (n = 45; see Supplementary material) included: participant demographics (n = 14); caregiver experiences with socialisation (n = 8); information about participant’s cats (n = 13); where caregivers receive socialisation information (n = 8); and ratings of various aspects of socialisation (n = 2). Participant demographic questions included age, gender identity, US state/territory of residence, past cat-related work experience, and self-rating of socioeconomic status. Given the broad range of income and cost of living throughout the US, the MacArthur Scale of Subjective Social Status (SSS; developed by Adler *et al.* 2000) was used to measure socioeconomic status. This scale asks participants to rate themselves out of ten rungs on a ladder, where they think they belong; ten indicates the most well off in their community and one indicates those who have the least. This scale is intended to assess where participants feel they stand financially in their community and been shown to be more predictive of a variety of measures of psychological well-being (Adler *et al.* 2000). Cat ownership questions included ownership of kittens and/or adult cats, the number of cats currently owned as the primary caregiver, and frequency (always, often, sometimes, rarely, never) of various cat behaviour issues.

The second section of the questionnaire asked if the participant had heard of kitten and adult cat socialisation programmes before, and whether they had attended a socialisation programme with a past pet. Questions asked where participants had received information on cat and/or kitten socialisation (e.g. internet, animal shelter, breeder, veterinarian, pet store), where past and current cats or kittens had been obtained (i.e. animal shelter, breeder, pet store, bred at home, gifted from friend/family, found on the street, other), and age of cat/kitten at the time of acquisition from each selection. Based on where they indicated they had obtained a past cat/kitten, they were asked to indicate socialisation information provided by various pet care professionals (i.e. shelter staff, breeders, pet store staff) and all respondents were asked to indicate socialisation information provided by their veterinarian. Types of socialisation information listed were: training to respond to commands; playtime with other kittens; interaction with animals other than cats; interaction with new people; education about common behavioural issues; getting kittens comfortable with nail trims; getting kittens comfortable with handling; carrier training; leash/harness training; and ‘other’ in which participants were able to input any additional aspect not mentioned above. Response options included: yes, no, not sure/cannot remember.

Participants were asked if they had taken part in a kitten socialisation programme in the past (yes, no). If participants selected yes, they were shown additional questions about the programme (n = 4) including: the approximate length of the programme (in hours); programme format (online asynchronous or synchronous, in-person, both in-person and online); and location (veterinary clinic, shelter facility, breeder’s house/facility, local university, private behaviour consulting business, other). Finally, participants were asked to indicate (yes, no, unsure/do not remember) aspects included in the programme (i.e. body language/behaviour, training cat to respond to commands, playtime with other kittens, kitten interaction with new people, etc).

Participants were asked if they would enroll in a future kitten, and adult cat socialisation programme (yes, no, unsure). If yes was selected, participants were shown a question asking to select reasons (e.g. improve cat/kitten’s co-operation at the veterinary clinic, improve cat/kitten’s health, getting help for existing cat/kitten behaviour problems, reducing future cat behaviour problems) they want to enroll. If no was selected, participants were shown a question asking to select reasons (e.g. price, transportation challenges, travel with cat/kitten is too stressful, no need for cat/kitten to be exposed to new animals) they would not want to enroll. If unsure was selected, participants were shown both questions asking to select reasons that they would and would not want to enroll.

Participants were then asked to rate the importance (not at all important, somewhat important, moderately important, very important, extremely important) of various aspects of a socialisation programme. Aspects mirrored those in the earlier question regarding information they have received from veterinarians about socialisation, with the addition of: education about cat body language; litter-box training; and homework (tasks for the owner to complete at home). Participants were also asked to rate the importance (5-point Likert scale) of socialisation during a cat’s various life stages: 2–9 weeks, 10 weeks–1 year old, over 1 year–6 years old, 7–10 years old, 11+ years old. These age ranges were chosen based on the AAHA/AAFP Feline Life Stages guidelines (Quimby *et al.* 2021), with the addition of the purported kitten socialisation period, 2–9 weeks (Karsh & Turner 1988; McCune 1995).

Finally, participants indicated using an open-ended text box numerical entry question, how much they would be willing to pay for a hypothetical kitten socialisation programme. This hypothetical programme (Landsberg *et al.* 2013) included a 1-h in-person class each week for three weeks. During this programme kittens are introduced to unfamiliar kittens and people, and cat owners are given homework to complete in between classes.

The questionnaire was advertised through snowball sampling via postings on social media sites (i.e. Facebook and Twitter/X), as well as an article advertising recruitment in a US-based online news outlet. Data collection took place from July 6^th^ to July 8^th^, 2022.

### Data analysis

Analyses were conducted using jamovi (v 2.4) and Rstudio (v 4.3.1, R Core Team, Vienna, Austria) statistical software. All responses were retained for participants meeting the inclusion criteria and reaching the end of the survey, indicated by answering the final question regarding the price they were willing to pay for a hypothetical kitten socialisation class. Descriptive statistics (frequencies, percentages) were generated for each question. For ease of analyses, state of residence was grouped based on geographic region as determined by the United States Census (Northeast, South, Midwest, and West). Gender was also grouped into three categories (man, woman, non-binary/third gender), with responses to the ‘other’ category being grouped with either ‘non-binary/third gender’ or omitted due to supplying an answer other than their gender identity. Additionally, the SSS scale responses were grouped into three categories, with 1–4 indicating lower SSS, 5–7 indicating middle SSS, and 8–10 indicating upper SSS. To create a binary variable depicting the presence or absence of behaviour problems in their current cat, the ‘never’ and ‘rarely’ responses were grouped as absent, while ‘sometimes’, ‘often’, and ‘always’ were grouped as present. Finally, responses on willingness to pay for a hypothetical kitten socialisation programme were grouped into quartiles: < $US45, $US45-74, $US75–99, and $US100+.

### Logistic regression

A multinomial logistic regression model was used to assess the relationship between the outcome variable ‘willingness to enroll in a future kitten socialisation programme (yes, no, unsure)’ and explanatory variables including participant demographics (age, SSS, gender, geographic area, region of the United States, and work history), presence or absence of behaviour problems in current cat, and the monetary amount participants were willing to pay for a future kitten socialisation programme. Given the large number of explanatory variables, and the aim of creating a parsimonious model, bivariate analyses (outcome variable with each explanatory variable) were performed using a liberal *P*-value of *P* ≤ 0.20 to assess eligibility for inclusion into the multivariable model (Dohoo *et al.* 2014). Model building used backwards elimination in which all eligible explanatory variables were included and those with the most non-significant (*P* > 0.05) contribution to the outcome were taken out one-by-one until only variables which were significant (*P* < 0.05) remained in the model. Plausible interactions based on prior predictions were also included in the model building process. To reduce the potential for Type 1 errors, a holm-bonferroni adjustment was used when multiple comparisons included four or more pairs. Model fit was assessed using AIC/BIC with a lower value preferred.

## Results

### Participants

A total of 2,238 responses were analysed, and the number of responses varied among the questions. Participants frequently indicated they were 35–44 years old (26.3%; Table 1), women (74.7%), and did not have children (79.8%). Participants frequently reported living in the western (35.2%) or southern (29.2%) regions of the US, indicated being in the middle SSS category (60.3%), had owned a dog at some point in their life (71.6%), and had not worked with cats in a professional setting in the past (72.9%). The behaviour problem most frequently reported being present in their current cat was excessive vocalisation (68.7%; Table 2). Of those who indicated working with cats in a professional setting (n = 607), participants most frequently indicated working in a shelter (43.3%), as kennel staff (30.8%), in the veterinary field (30.3%), and/or as a cat sitter (28.0%). Those who worked with cats in a professional setting indicated working in the position(s) for 1–5 years (42.8%), 6–15 years (22.4%), less than 1 year (17.8%), or 16+ years (17.0%).

**Table 1.** Demographic descriptive information for US cat owner participants (n = 2,238) who completed an online questionnaire on their attitudes towards kitten and adult cat socialisation programmes

Characteristic	%	n
**Age**		
18–34	19.9	445
35–44	26.3	588
45–54	21.6	483
55–64	18.3	410
65+	13.9	312
**Gender**		
Woman	74.7	1671
Man	21.4	480
Non-Binary/Third Gender	2.8	63
**Children**		
Yes	19.7	440
No	79.8	1787
**Owned a dog**		
Yes	71.6	1603
No	28.3	634
**Geographic area**		
Urban	24.6	550
Suburban	58	1299
Rural	17.4	389
**Subjective social status**		
1–3 (Low)	13	291
4–6 (Middle)	60.3	1350
7–10 (High)	26.7	597
**Region**		
Northeast	14.3	320
Midwest	21.3	476
South	29.2	654
West	35.2	788
**Work experience**		
Yes	27.1	607
No	72.9	1631
**Cats**		
1	28	632
2	38	850
3	13.4	301
4+	20.3	455
**Location**		
Shelter	72.4	1621
Breeder	10.2	228
Pet Store	7.6	171
Bred at Home	6.4	143
Gifted	30.6	685
Found/picked up from street	58.3	1305

**Table 2.** Frequency of various behaviour problems reported to occur by cat owner participants’ current cat (n = 2,238), presented as frequency(%)

Behaviour problem	Always	Often	Sometimes	Rarely	Never
Aggression	8(0.4)	118(5.3)	640(28.6)	1018(45.5)	454(20.3)
GI & ingestive diseases	24(1.1)	249(11.1)	788(35.2)	801(35.8)	376(16.8)
Separation anxiety	59(2.6)	262(11.7)	701(31.3)	708(31.6)	508(22.7)
Urinating/defaecating outside litter-box	21(0.9)	73(3.3)	290(13)	705(31.5)	1149(51.3)
Excessive vocalisation	169(7.6)	620(27.7)	747(33.4)	546(24.4)	156(7)
Excessive night-time activity	56(2.5)	223(10)	695(31.1)	981(43.8)	283(12.6)
Unwanted behaviour	72(3.2)	246(11)	685(30.6)	890(39.8)	345(15.4)
Destructive behaviour	31(1.4)	279(12.5)	836(37.7)	844(37.7)	248(11.1)
Fears/phobias	67(3)	301(13.4)	806(36)	753(33.6)	311(13.9)
Abnormal behaviour	15(0.7)	77(3.4)	299(13.4)	811(36.2)	1036(46.3)

Participants frequently indicated owning one (28.2%) or two (38.0%) cats, and most indicated adopting a past or current cat from a shelter (72.4%) or finding their cat on the street (58.3%). Most participants who indicated adopting from a shelter (n = 1,622), reported adopting a cat/kitten between 10 weeks to < 1 year old (56.7%), 1–6 years old (42.1%), 0–9 weeks old (29.9%), and/or ≥ 7 years old (7.9%). Those who indicated purchasing a cat/kitten from a breeder (n = 249) and/or pet store (n = 188), most often indicated purchasing their kitten between 10 weeks–1 year old (breeder: 65.8%; pet store: 52.9%) followed by 0–9 weeks old (breeder: 32.9%; pet store: 36.5%), 1–6 years old (breeder: 10.5%; pet store: 20.0%) and/or ≥ 7 years old (breeder: 0.0%; pet store: 1.2%).

Participants’ interest in a future socialisation programme varied by age of cat; participants most frequently indicated interest in a future programme (kitten: 50.4%; adult: 38.6%), while some were unsure (kitten: 30.9%; adult: 36.8%), or indicated no interest (kitten: 18.8%; adult: 24.6%). Participants were willing to spend a mean (± SD) of $US92.73 (± 83.24; min: $US0, max: $US1,000) on a proposed hypothetical kitten socialisation programme.

### Information about socialisation

Most participants indicated they had never heard of kitten or adult socialisation programmes (kitten: 69.3%; adult: 73.5%), while some had heard of these programmes (kitten: 26.6%; adult: 21.9%) or were unsure (kitten: 4.0%; adult: 4.5%).

Participants indicated getting their information about socialisation from the internet (52.3%), veterinarians (31.2%), books (20.6%), animal shelters (20.3%), professional behaviourists (6.3%), pet stores (4.3%), breeders (2.1%), and/or groomers (0.6%).

**Figure 1.**
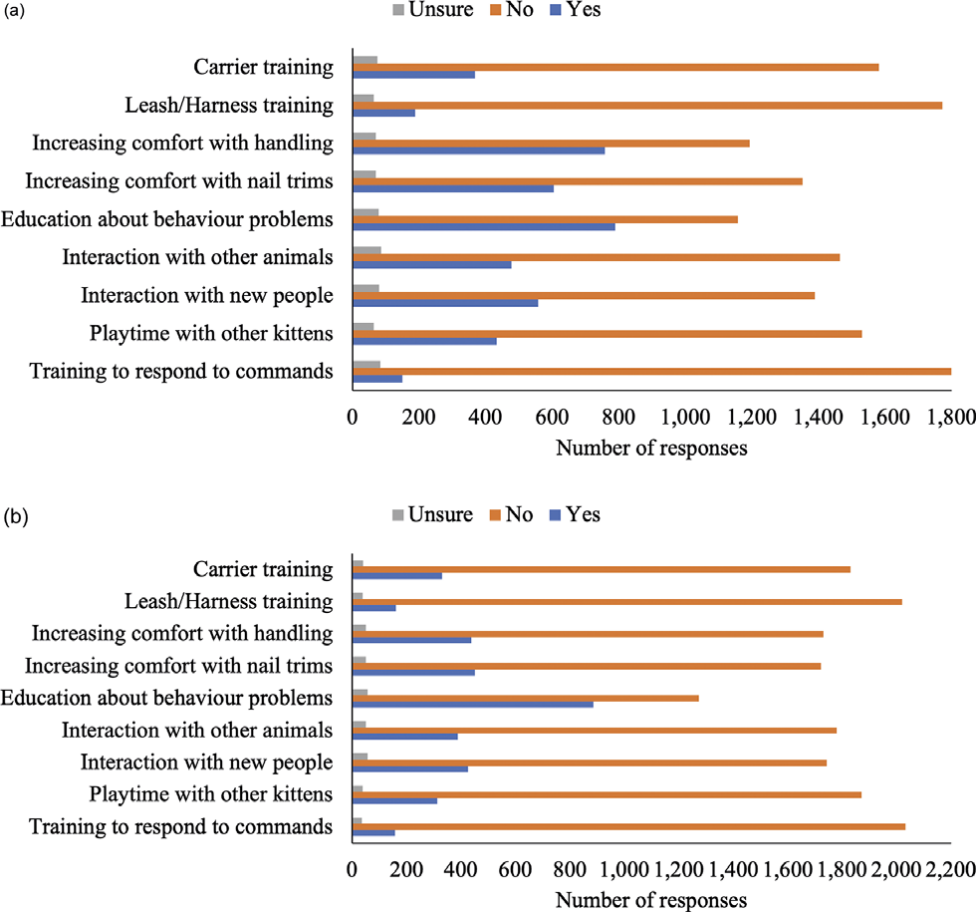
Cat owner reports of information received regarding different aspects of (a) kitten (n = 2,021–2,033) and (b) adult cat (n = 2,200–2,225), socialisation information from their veterinarian. Total participant responses varied for each question.

**Figure 2.**
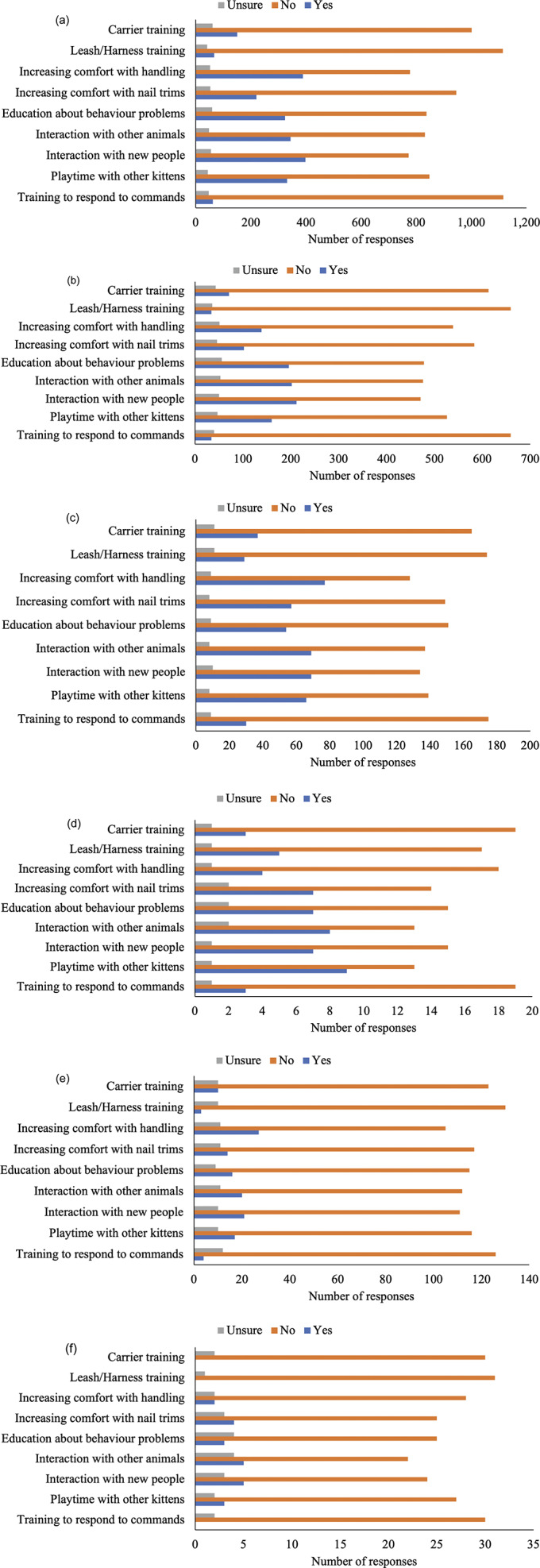
Cat owner reports of information received regarding different aspects of socialisation upon adoption or purchase from an (a) shelter - kitten (n = 1,213–1,226), (b) shelter - adult cat (n = 727–733), (c) breeder - kitten (n = 213–214), (d) breeder - adult cat (n = 23–24), (e) pet store - kitten (n = 140–143) and (f) pet store - adult cat (n = 31–32). Total participant responses varied for each question.

Next, questions asked about socialisation information received during kitten/adult cat adoption and during veterinary visits. Information participants (n = 2,238) received from veterinarians about various socialisation topics for kittens and adult cats are presented in Figures 1(a) and (b). Socialisation information participants received from shelter staff, breeders, and pet stores during a kitten and/or adult cat adoption are presented in Figures 2(a)–(f). In summary, most respondents (> 50%) indicated not receiving information on each aspect of socialisation from veterinarians, shelter staff, breeders, and/or pet stores.

**Figure 3.**
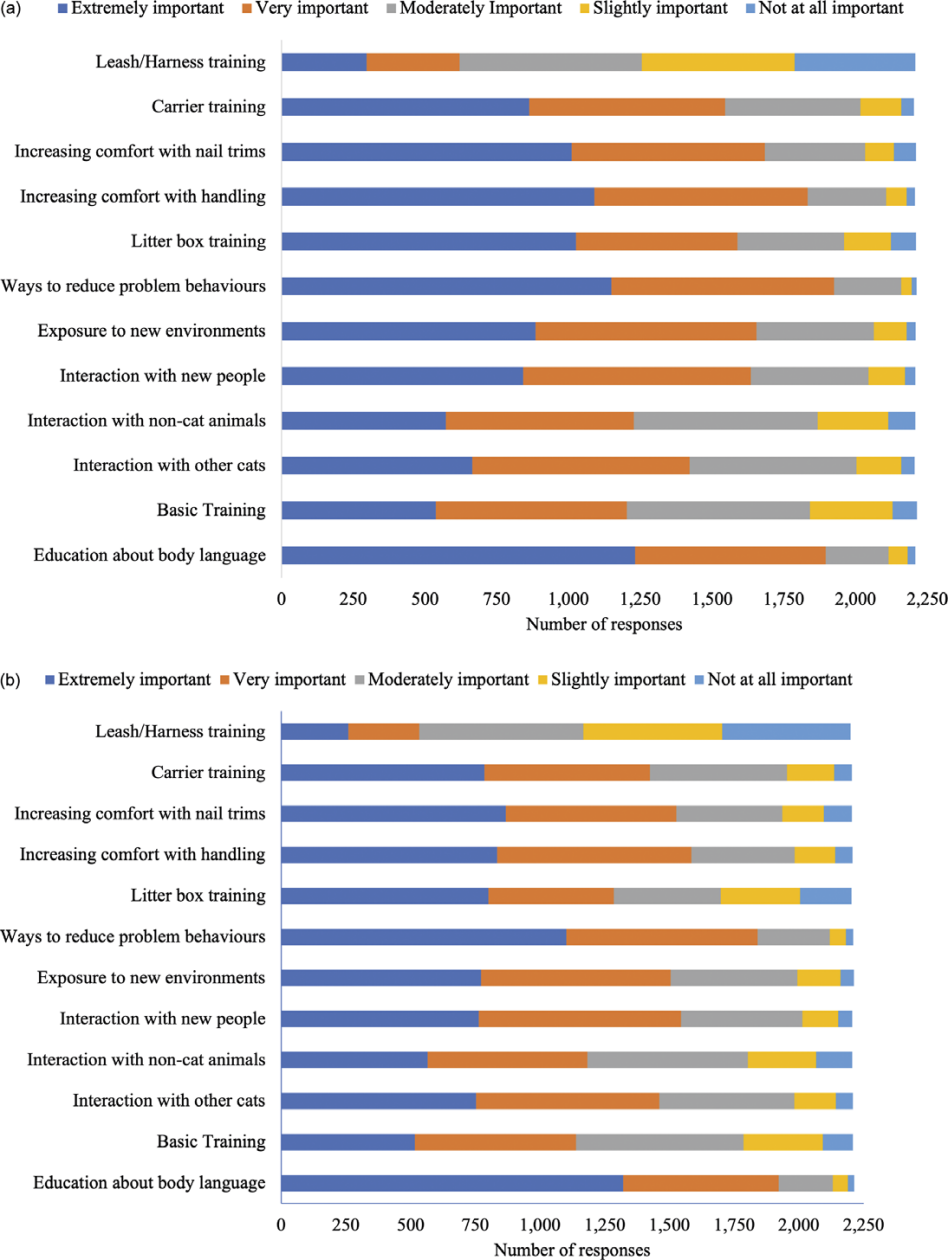
Cat owner ratings regarding the importance of various components of socialisation programmes for (a) kittens (n = 2,203–2,213) and (b) adult cats (n = 2,199–2,214). Total participant responses varied for each item.

Aspects of socialisation ranked by participants as being the most important (> 80% indicated very or extremely important) to include in a socialisation programme for kittens were: ways to reduce problem behaviours (87.0%; Figure 3[a]); education about body language (85.8%); and getting kittens comfortable with handling (83.1%). For adult cats, participants ranked the most important (> 80% indicated very or extremely important) aspects of socialisation programmes as education about body language (86.8%; Figure 3[b]), and ways to reduce behaviour problems (83.3%).

### Enrolling in socialisation programmes for kittens and/or adult cats

**Figure 4.**
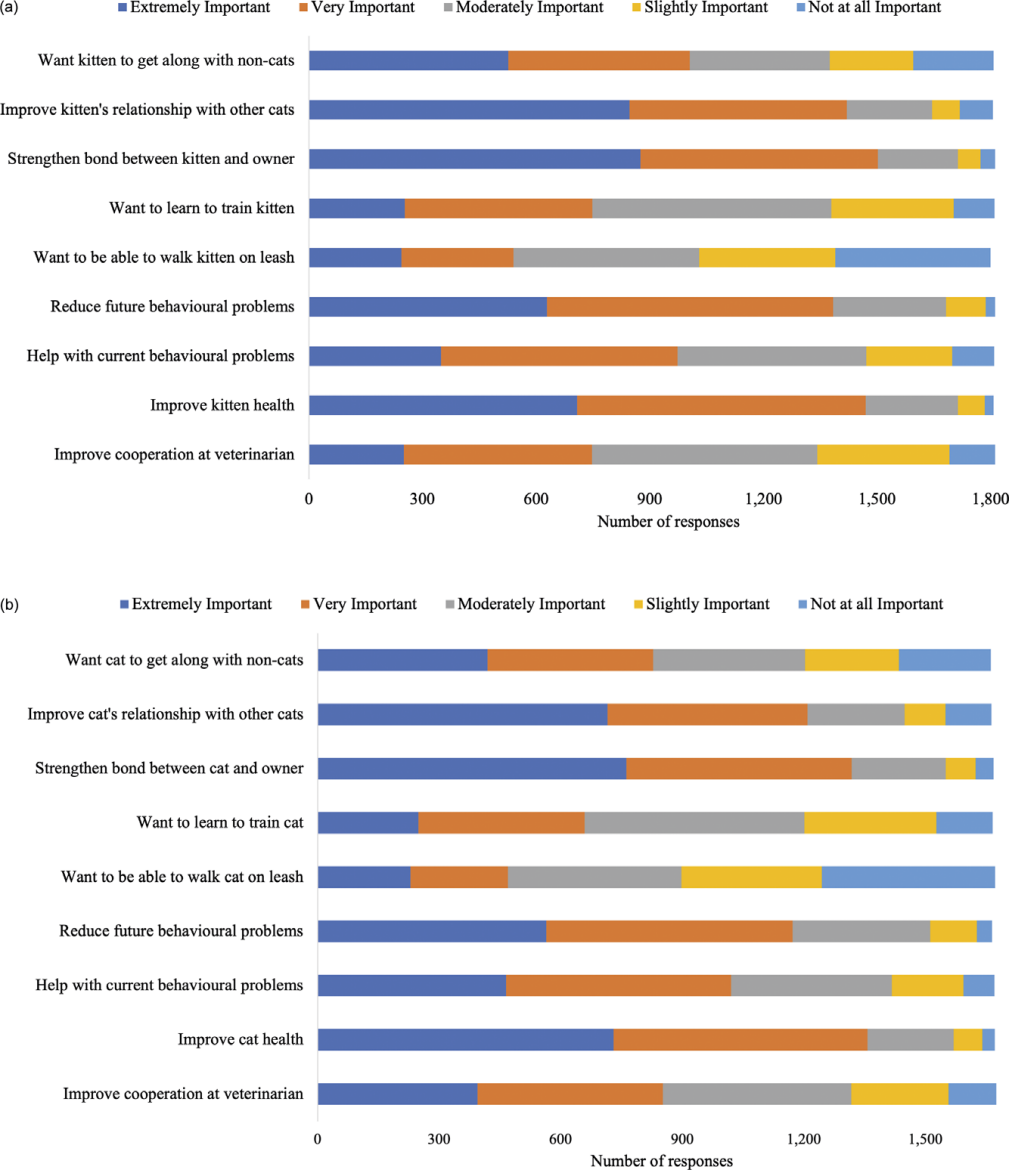
Cat owner ratings of importance regarding potential reasons to enroll in a future (a) kitten (n = 1,799–1,811) or (b) adult cat (n = 1,661–1,675) socialisation programme. Total participant responses varied for each item.

Aspects rated as the most important (> 75% indicated very or extremely important) reason for their decision to enroll in a future socialisation kitten and/or adult cat programme were strengthening the bond between the kitten/adult cat and the owner (kitten: 83.1%; Figure 4[a]; adult cat: 79.0%; Figure 4[b]) as well as improving kitten/adult cat health (kitten: 81.4%; adult cat: 81.2%).

**Figure 5.**
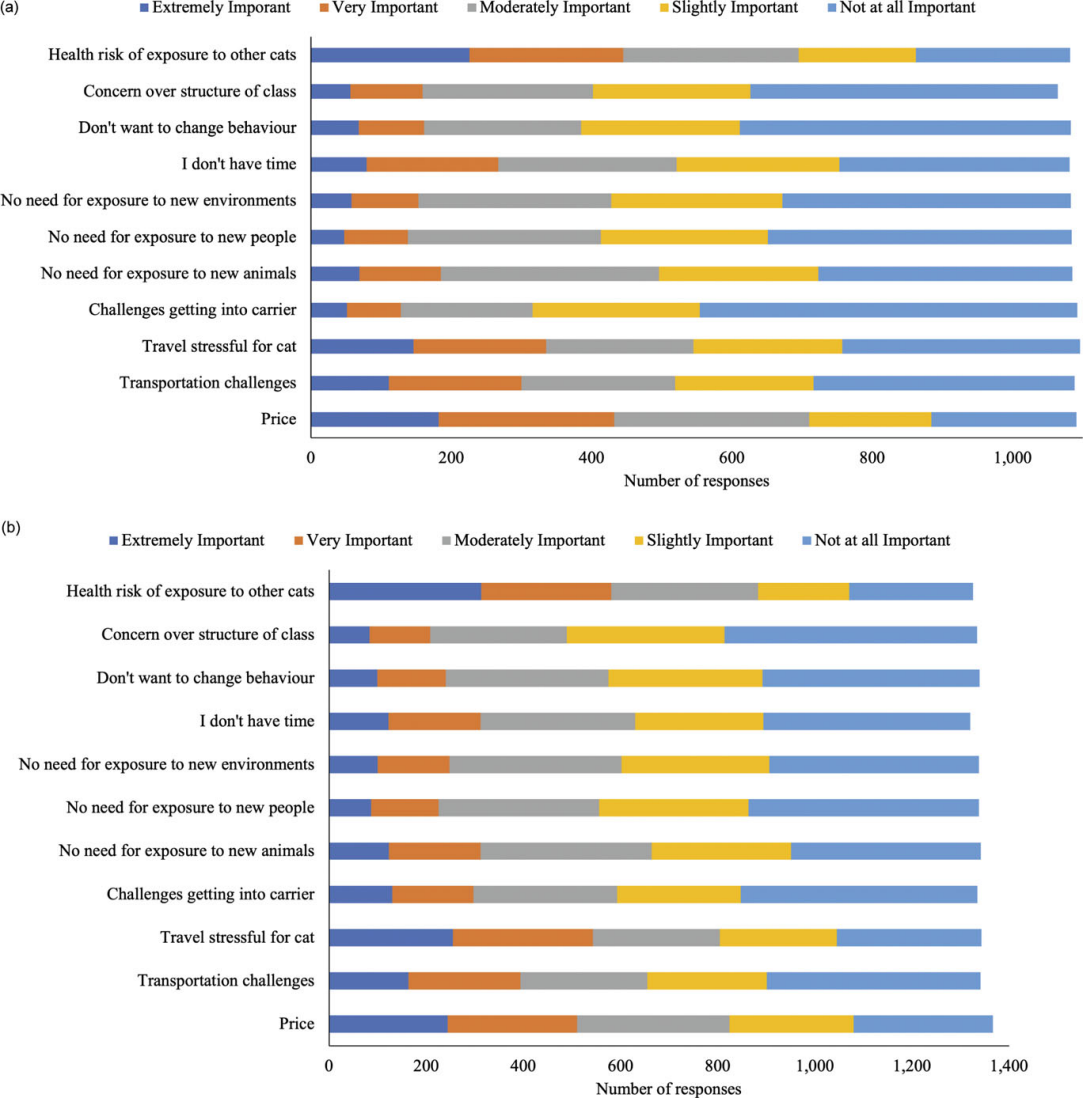
Cat owner ratings of the importance of reasons not to enroll in a future (a) kitten (n = 1,064–1,096) or (b) adult cat (n = 1,320–1,367) socialisation programme. Total participant responses varied for each item.

The reason most frequently rated as very or extremely important for participants *not* wanting to enroll in a future kitten and/or adult cat socialisation programme was health risks related to exposure to other kittens/cats (kittens: 41.1%; Figure 5[a]; adult cats: 43.8%; Figure 5[b]). Price was also frequently listed as an important reason not to enroll in a future kitten socialisation programme (39.6%), and concerns about travel-related stress for adult cats (40.4%) was another reason given frequently.

### Previous experience with kitten socialisation programmes

Thirty-two respondents indicated having participated in a kitten socialisation programme in the past, either in person (50.0%), online (synchronous: 6.3%; asynchronous: 25.0%) or both in-person and online (18.8%). These programmes were conducted in a shelter (43.8%), veterinary clinic (18.8%), private behaviour consulting business (12.5%), breeders house (6.3%), university (3.1%), or other (e.g. cat rescue, online conference, library; 15.6%) and took an average of 8 h 59 min (min: 1 h, max: 36 h) to complete.

Those who had participated in a previous kitten socialisation programme (n = 32) indicated that ‘getting kittens used to handling’ was included in every programme (100.0%). Common components of socialisation programmes included lessons on cat body language (yes: 81.3%; no: 15.6%; unsure: 3.1%), playtime with other kittens (yes: 90.6%; no: 9.4%), kitten interaction with new people (yes: 87.5%; no: 12.5%), interaction with animals other than cats (yes: 68.8%; no: 25%; unsure: 6.3%), education about common behavioural problems (yes: 78.1%; no: 21.9%), getting kittens comfortable with nail trims (yes: 62.5%; no: 34.4%; unsure: 3.1%), carrier training (yes: 59.4%; no: 31.3%; unsure: 9.4%), and homework (yes: 65.5%; no: 21.9%; unsure: 12.5%). Components not commonly included in socialisation programmes were leash/harness training (yes: 34.4%; no: 56.3%; unsure: 9.4%), and training to respond to commands (yes: 40.6%; no: 50.0%; unsure: 9.4%).

### Interest level in future kitten socialisation programme

Factors influencing interest in participating in a future kitten socialisation programme were participant age, gender, and geographic area (rural, urban, suburban), whether participants had attended a socialisation programme in the past for any pet, the presence of aggression in current cat, ratings of importance of socialisation at different life stages, and the amount participants were willing to pay for a hypothetical socialisation programme. Table 3 reports regression model results with associated ORs, 95% CIs, and *P*-values.

**Table 3.** Multinomial logistic regression results showing significant predictors of cat owner participant interest (yes, no, unsure) in a future kitten socialisation programme (n = 2,214), presented with P-values, odds ratios, and 95% confidence intervals

Variable	Yes vs No		Yes vs Unsure		No vs Unsure	
	OR(95% CI)	*P*-value	OR (95% CI)	*P*-value	OR (95% CI)	*P*-value
**Attended socialisation class for any pet**						
Yes vs No	1.87(1.33, 2.61)	< 0.001	1.29(1.02, 1.64)	0.036	0.69(0.49, 0.99)	0.041
**Importance at 2–9 weeks**						
Extremely important vs Very important	-	-	1.44(1.12, 1.86)	0.037^*^	-	-
Extremely important vs Moderately important	1.50(1.03, 2.18)	0.027^*^	1.81(1.34, 2.45)	<0.001^*^	-	-
Extremely important vs Slightly important	-	-	2.19(1.43, 3.37)	0.002^*^	-	-
Extremely important vs Not at all important	6.06(2.61, 14.06)	<0.001^*^	3.86(1.70, 8.76)	0.006^*^	-	-
Very important vs Not at all important	4.48(1.88, 10.67)	0.004^*^	-	-	-	-
Moderately important vs Not at all important	4.05(1.66, 9.89)	0.015^*^	-	-	-	-
**Aggression in current cat**						
Present vs Absent	-	-	1.33(1.08, 1.64)	0.007	-	-
**Amount willing to pay for socialisation class**						
$US100+ vs $US45–74	1.78(1.27, 2.49)	< 0.001	1.82(1.43, 2.33)	<0.001	0.34(0.24, 0.48)	<0.001
$US100+ vs <$US45	5.45(4.00, 7.43)	< 0.001	1.87(1.42, 2.47)	<0.001	-	
$US75–99 vs <$US45	4.93(3.07, 7.93)	< 0.001	-	-	0.29(0.18, 0.48)	<0.001
$US45–74 vs <$US45	3.06(2.18, 4.30)	< 0.001	-	-	0.34(0.24, 0.47)	<0.001
**Age**						
18–34 vs 35–44	-	-	1.54(1.15, 2.07)	0.036	-	-
18–34 vs 55–64	-	-	1.62(1.17, 2.25)	0.03^*^	-	-
**Gender**						
Woman vs Man	1.61(1.21, 2.14)	<0.001	-	-	0.68(0.50, 0.91)	0.01
Woman vs Non-binary/third gender	-		-	-	3.80(1.12, 12.97)	0.033
Non-binary/third gender vs Man	4.73(1.38, 16.14)	0.013	-	-	0.26(0.08, 0.09)	0.007
**Geographic area**						
Urban vs Rural	1.72(1.18, 2.52)	0.005	-	-	-	-
Suburban vs Rural	1.48(1.07, 2.03)	0.017	-	-	-	-

* Adjusted *P*-value using holm-bonferroni

## Discussion

Approximately half of the study participants indicated interest in a future kitten socialisation programme, however less than 2% indicated ever having participated in a past programme. It is likely that few participants have experience with kitten socialisation programmes due to a lack of knowledge, given that most indicated they had never heard of a socialisation programme for kittens. Nevertheless, participants indicated being willing to spend an average of $US92 on a standard three-week kitten socialisation programme, suggesting participants recognised the potential value of these programmes.

The most frequent reason participants gave for not wanting to enroll their kitten in a socialisation programme was health risks associated with exposure to other kittens. There is always a risk of pathogen exposure when pets are exposed to conspecifics or other species. To minimise risks, veterinary recommendations suggest kittens should receive their first set of core vaccinations at least 14 days before the first class and should not show evidence of illness or disease (Hetts *et al.* 2004; Overall *et al.* 2005; Crowell-Davis 2007; Landsberg *et al.* 2013; AVMA 2024; Squires *et al.* 2024). These guidelines also suggest that the benefits of socialisation programmes outweigh disease risks associated with enrolling pets into programmes prior to receiving their full set of core vaccines. Classes conducted in a controlled environment, such as a disinfected indoor space, may introduce fewer disease risks than a less-controlled environment such as an outdoor green space where there may be increased risks of pathogen exposure (Stull *et al.* 2016). In puppies, one study suggests that those vaccinated with the first round of parvovirus vaccine who had attended a socialisation programme, were at no greater risk of parvovirus infection than those who had not attended classes (Stepita *et al.* 2013), suggesting that these classes do not pose excess disease risks. However, the purported sensitive period of socialisation in kittens ends earlier than in puppies (2–7 or 9 weeks versus 3–12 or 14 weeks, respectively; Freedman *et al.* 1961; Scott & Fuller 1965; Karsh & Turner 1988; McCune 1995). This means the sensitive period of socialisation has likely passed by the time kittens receive their first set of vaccines. Since many shelters do not adopt out kittens younger than eight weeks of age, kitten fosters and shelter staff should be targeted to implement various aspects of socialisation during the sensitive period of socialisation. Another potential reason participants indicated health risks as a top reason to not enroll a kitten in a socialisation programme, may be related to this research being conducted during the COVID-19 pandemic. Thus, respondents may have been more cautious of disease risks at the time of data collection.

Accessing kitten socialisation programmes may be a significant barrier for owners who are interested in enrolling, as in-person programmes may be difficult to find. Offering virtual classes may help reduce barriers related to transportation and distance that may interfere with participants’ ability to access these programmes. Alternatives to in-person socialisation programmes include synchronous and asynchronous online classes. A virtual setting may allow a greater focus on education, for example, education on cat body language and behaviour. This is important, given that educating owners about normal cat behaviour may help reduce reports of misbehaviour in pets (Grigg & Kogan 2019).

Notable factors that were predictive of interest in a future kitten socialisation class were participant gender, the presence of aggression in their current cat, participation in a past programme with any pet, and geographic area (urban, suburban, rural). Our results suggest women are more likely than men and non-binary individuals to enroll in a future kitten socialisation programme. Previous studies which have assessed cats in a veterinary clinic often report a majority of participating owners as women (Shaw *et al.* 2012; Boone *et al.* 2023), which may suggest that women are more active participants in their cat’s care compared to men. Women have a long history of close attachments to cats (Frasin 2022) and have been found to speak and interact with their cats more than men do (Mertens & Turner 1988; Mertens 1991). One study suggests that 79% of participants categorised pet cats as either ‘family’ or ‘children’ in the home (Bouma *et al.* 2021) and as women are typically expected to fulfill a more caregiving role in the family, it may be that they are more interested in participating in cat care activities such as socialisation classes.

Participants attending a past socialisation class with any animal were also more likely to indicate interest in enrolling in a socialisation programme compared to those that had not attended a class before. These results are encouraging and suggest that pet owners that have attended classes in the past recognise the benefits of such programmes. Additionally, those in current possession of a cat with an aggression-related behaviour problem were more interested in enrolling in a future socialisation programme. These participants were more likely to respond ‘yes’ as opposed to ‘unsure’, for enrolling in a future programme. This suggests that the presence of a serious cat behavioural issue, such as an aggression-related problem, may motivate cat owners to participate in a socialisation programme in an attempt to manage this problem. Aggression towards people or animals is often listed as a primary reason for relinquishment or return to shelters (Salman *et al.* 2000; Casey *et al.* 2009) and is a primary concern for new adopters (O’Connor *et al.* 2017). Interestingly, three other cat behaviour problems were frequently reported by participants, although these were not associated with interest in enrollment in a future kitten socialisation programme: excessive vocalisation; destructive behaviour; and fears/phobias. Previous research suggests a high prevalence of these cat behaviours in the home (Morgan & Houpt 1989; Heidenberger 1997; Grigg & Kogan 2019), and reports that many cat owners (> 45%; n = 547) are not ‘bothered at all’ by their cat’s excessive vocalisation, destructive behaviours, or fear and anxiety (Grigg & Kogan 2019). However, cat owners are recommended to take a proactive rather than a reactive approach to behaviour problems by enrolling in a socialisation programme soon after adoption.

Individuals who live in urban and suburban geographic areas were more likely to indicate interest in enrolling in a future kitten socialisation programme, compared to those living in rural environments. This may point to cultural differences that exist in rural regions of the US regarding the role of cats in society. It may be that barn cats (semi-feral cats who live and hunt on a farm) are more common in rural areas, with farmers specifically seeking out and adopting these animals with the aim of reducing the rodent population on their property (‘Barn & Garden Cat Adoptions’ undated; Overstreet undated; Kitts-Morgan *et al.* 2015). Thus, perceiving cats as working animals may reduce interest in a socialisation programme. However, research elucidating differences in attitudes towards cat ownership between rural, suburban, and urban areas is needed.

Despite most participanting cat owners indicating not being educated about kitten socialisation by pet care professionals (veterinarians, shelter staff, etc), many indicated that various aspects of socialisation were very or extremely important. Individuals emphasised the importance of education about body language and problem behaviours, as well as getting kittens comfortable with handling. These three aspects of socialisation are routinely recommended for inclusion in socialisation programmes for kittens (Overall *et al.* 2005; Seksel & Dale 2012; Landsberg *et al.* 2013; Blackwell 2022). Other routinely recommended practices had less owner interest, such as training to respond to commands and interaction with other cats. This may be related to commonly believed myths that cats are ‘asocial’ and/or ‘untrainable’ (Overall *et al.* 2005; Seksel & Dale 2012; Blackwell 2022). However, domestic cats are well established as being social (Suchak *et al.* 2016; Vitale 2022), and research suggests training programmes to be beneficial for cats in the shelter (Bollen 2015; Kogan *et al.* 2017; Grant & Warrior 2019), although more research is needed regarding the appropriate age to begin training kittens to respond to commands (Graham *et al.* 2024b).

In lieu of information from pet care professionals, participants indicate receiving most of their information on socialisation for kittens from the internet, with some referencing internet/tv personalities such as Jackson Galaxy (n = 20). Although many reputable online socialisation resources exist (e.g. AVMA undated; Ellis undated), caregivers may not know where to find reputable sources and their search may lead to misinformation. It is recommended that pet care professionals educate kitten and adult cat owners on socialisation and where to access reliable information on socialising their new pet. Veterinary recommendations surrounding socialisation are centered around addressing behaviour concerns of the cat owner (AAHA-AVMA 2011), although some veterinary resources offer explicit recommendations for when and how best to socialise kittens in early life and up to one year old (Overall *et al.* 2005; AVMA 2015, undated). For example, a number of socialisation recommendations suggest encouraging owners to use positive reinforcement and allow the cat to progress at its own pace (AVMA undated). The American Association of Feline Practitioners (AAFP) emphasises exposure to new environments and people, as well as training using positive reinforcement (Overall *et al.* 2005). Research suggests that owners who were provided advice for preventing behaviour problems by a veterinary behaviourist at the first appointment were less likely to report behaviour problems ten months later than those who did not receive advice (Gazzano *et al.* 2015). This finding suggests that education provided by veterinarians at a kitten’s first veterinary appointment may benefit the human-animal bond as well as the cat’s success in the home. Although owner education on cat and kitten behaviour is important, reports suggest veterinarians receive little education on cat behaviour and welfare during veterinary school (Shivley *et al.* 2016). In a nationwide survey of over 1,000 veterinarians, more than 90% reported not feeling that they had received sufficient cat behaviour training at veterinary school (Kogan *et al.* 2020), and another survey of graduating veterinary students showed that less than 30% of 366 students surveyed indicated feeling prepared by their clinical behaviour curriculum (Calder *et al.* 2017). This reflects the need for veterinary school curricula to provide an increased emphasis on cat behaviour and welfare, as well as best practice at-home management information.

Although veterinary recommendations include kitten socialisation classes for preventing behaviour problems in adulthood (Hetts *et al.* 2004; Crowell-Davis 2007; Seksel 2008; Landsberg *et al.* 2013), no research has examined the impact of these programmes on current and future behaviour and health outcomes, nor on owner knowledge and management of their cat in the home. Elsewhere, research has assessed the impact of socialisation programmes on puppy health and behaviour. Retrospective analyses suggest that dogs reported to have attended puppy socialisation classes in the past show a decrease in reactivity to unfamiliar humans and dogs outside the household (Blackwell *et al.* 2008; Casey *et al.* 2014), as well as reduced fear-related responses to loud noises such as thunderstorms or vacuum cleaners (Cutler *et al.* 2017). Other research incorporating owner questionnaire pet assessments (C-BARQ: Hsu & Serpell 2003; Puppy Walker Questionnaire: Asher *et al.* 2013), in which puppies either took or abstained from a socialisation class, has found that puppies who attended a socialisation class had lower levels of reported touch-sensitivity and non-social fear (González-Martínez *et al.* 2019), as well as reduced separation-related behaviours and general anxiety scores (Vaterlaws-Whiteside & Hartmann 2017). Further, Vaterlaws-Whiteside and Hartmann (2017) suggest that puppies who had participated in additional socialisation experiences compared to puppies who had not, had more desirable behavioural responses when exposed to various types of stimuli, including unfamiliar people and objects. However, further research is needed to assess the impact of socialisation classes for kittens and elucidate the aspects of these programmes which carry the most benefit for the cat and the owner.

Aside from veterinary guidelines and best practice statements on kitten and puppy socialisation, to the authors’ knowledge, no other socialisation guidelines exist for non-veterinarians such as shelter staff or pet store staff. However, many shelter facilities may have staff with a special interest in behaviour and socialisation. For example, some shelter facilities have staff members who run socialisation classes or specialise in socialising fractious kittens and/or shy adult cats. These individuals may have access to reputable resources and may play a key role in educating other staff members and the public (i.e. new kitten adopters) on socialisation topics. Many animal shelter organisations collaborate with local pet stores and may house kitten/adult cats at pet stores to help increase adoption. Our survey did not differentiate between pet stores who collaborate with local shelters for increasing kitten/cat adoption and those that do not, which may be important for understanding how much socialisation information new kitten owners are provided. For example, some pet stores partner with local shelters to increase pet adoptions, providing information to new cat owners via their website which has a section dedicated to education on topics relevant to these individuals (i.e. tips on cat furniture, feeding, and helping a new cat adjust to its new home; Petco undated). Further research should assess how this information correlates with current recommendations. In addition, future work should attempt to compile socialisation information disseminated to new cat owners at adoption/purchase to better understand the information exchanged and how this aligns with current veterinary and behaviour expert socialisation recommendations.

Socialisation is often referred to as a life-long process (Hetts *et al.* 2004; AVMA 2015, 2024; Petak 2022), although little research has assessed the impact of continued socialisation on cat behaviour and health outcomes. To our knowledge, the current study is the first to ask cat owners their opinions on adult cat socialisation practices and programmes. Most participants indicated they were not interested in attending adult cat socialisation classes, with many reporting concern regarding health issues and indicating that travel would be stressful for their cat. Online socialisation classes aimed primarily at owner education may be a suitable alternative for owners who are particularly concerned with stress caused by travel. Caution should be noted for adult cat socialisation classes involving exposure to new environments and unfamiliar people, both of which have been associated with negative responses in adult cats (Carlstead *et al.* 1993; Slater *et al.* 2013). Thus, it may be beneficial to structure adult cat versus kitten socialisation programmes differently. For example, virtual programmes may be more reasonable for adult cats, as well as focusing more on owner education and improving the human-cat bond. Whereas either virtual or in-person programmes may be effective for kittens, with a focus on exposure to novel stimuli (e.g. new animals, people, environments) as well as owner education. Research is needed to assess the most effective way to implement adult cat versus kitten socialisation programmes, as well as components of these programmes that would be beneficial for each life-stage. Further research is also needed to produce effective, evidence-based protocols for various components of a socialisation programme, such as handling cats for nail trims.

### Study limitations

Some of the study questions relied upon participants remembering socialisation information provided to them during their cat’s adoption/purchase, and during their cat’s initial veterinary examination post-adoption. This information is vulnerable to recall bias as participants may only remember some or none of this information, rather than what they were told in entirety. In addition, most of our survey participants were women, those living in suburban environments, and those without children, which is not representative of all cat owners. However, a large percentage of woman respondents is common in online surveys (Sax *et al.* 2003). This represents a clear limitation, although it is not unique to our survey nor to cat survey research. In addition, other cat survey research similarly shows a large percentage of respondents indicating not living with children (Grigg & Kogan 2019; Khoddami *et al.* 2023). This may be due to this population of cat owners who have the time, or greater inclination, to complete questionnaires related to their cat(s). In addition, it is possible this questionnaire attracted a specific population of cat owners with particular interest in socialisation, and thus the data presented may not be representative all cat owners in general.

### Animal welfare implications

Kitten and cat socialisation programmes aim to increase owner education and promote practices for optimising cat health, welfare, behavioural management, and a strong human-cat bond. The current study provides information on aspects of kitten and adult cat socialisation deemed important by cat owners, elucidates reasons cat owners would or would not want to enroll in kitten and adult cat socialisation programmes, and highlights areas for future work. It is important to note that travel and exposure to new environments, people, and animals are inherent to kitten socialisation classes, and may result in excess fear and stress for some kittens and adult cats. Thus, the mode of the socialisation programme (i.e. in-person, virtual) should be considered and selected on an individualised basis to promote animal welfare.

## Supporting information

Link and Moody supplementary material Link and Moody supplementary material

## Supplementary material

The supplementary material for this article can be found at http://doi.org/10.1017/awf.2025.10013.
