# Supplementary material for: Socialising kitties: A quantitative survey of US cat owner attitudes towards kitten and adult cat socialisation programmes
Source: Anim Welf. 2025 Jun 23;34:e39. doi: 10.1017/awf.2025.10013 (PMC12277100; doi:10.1017/awf.2025.10013)
Supplement: Link and Moody supplementary material [file S0962728625100134sup001.pdf]

Socialising kitties: A quantitative survey of US cat owner attitudes towards kitten and adult cat socialisation programmes

Jennifer K Link, Carly M Moody https://orcid.org/0000-0003-3444-5932

The Animal Welfare Epidemiology Laboratory, Department of Animal Science, University of California, Davis, CA, USA
Author for correspondence: Carly M Moody, email: cmoody@ucdavis.edu

Supplementary material

<u>Survey</u>

Cat Caregiver Attitudes Towards Methods of Cat Socialisation

You are invited to join a research survey conducted by Jennifer Link (PhD student, Animal Behavior Graduate Group, UC Davis) and Dr. Carly Moody (Assistant Professor, Dept of Animal Science, UC Davis). The purpose of this study is to investigate cat caregiver attitudes towards methods of cat socialisation.

Benefit to Participant
Although there is no direct benefit to the individual participating in the study, the cat behaviour and care communities may benefit from this research by providing valuable insights about cat caregiver attitudes towards socialisation.

Participation and Withdrawal
Participation in this research is completely voluntary. Participants must be 18 years of age or over and currently be the primary caregiver for at least one cat. Participation should take approximately 15 minutes. If at any time during the completion of this survey participants feel uncomfortable or feel the need to stop, they are free to do so without penalty. Please contact Jennifer Link at jlink@ucdavis.edu with any concerns or questions you may have about participation or withdrawal from this survey.

Confidentiality
All information received will remain anonymous and will be kept confidential. Please do not put your name anywhere in this survey. Please note that confidentiality cannot be guaranteed while data are in transit over the internet.

You can help ensure your privacy by taking the following precautions to clear all private data from the computer you are using to respond to the survey:
-Clear the browsing history
-Clear the cache
-Clear the cookies
-Clear the authenticated session
-LOG OFF
- If you are using Internet Explorer, the first 4 steps can be accomplished by going into --Tools and selecting
-Delete Browser History. Your application may have a similar system.

To minimise the risks of breach of confidentiality, data will only be accessible by the research team at UC Davis. This study poses no known physical or psychological risks to participants.

Compensation
You will not be paid for taking part in this study.

Rights
You are not waiving any legal claims, rights, or remedies because of your participation in this research survey.

If you have questions about your rights as a research participant, please contact the University of California Davis, Institutional Review Board at 916 703 9158 or HS-IRBEducation@ucdavis.edu.

This research has been reviewed by the UC Davis Institutional Review Board (IRB ID:1883274-1). If you have any questions about this research, please feel free to contact Jennifer Link at jlink@ucdavis.edu Please save or print this page now if you would like a copy for your records. Note: Your responses will automatically be saved as you go, and you may go back and change a response at any time before submission. If you have read the above information and agree to take part in the research, please click on the arrow button below.

Do you currently own a cat?

○ Yes

○ No (skip to end of survey)

Do you currently live in the United States?

- ◯ Yes
- ◯ No (skip to end of survey)

How old are you?

▼ Under 18 ... 85 or older

With which gender do you most identify?

- ◯ Man
- ◯ Woman
- ◯ Non-binary / third gender
- ◯ Prefer to self describe ____
- ◯ Prefer not to say

Do you have any dependents (i.e. children) who live with you?

- ◯ Yes
- ◯ No
- ◯ Other ____
- ◯ Prefer not to say

What word would best describe the area in which you currently live?

○ Urban area (city center or metropolis)

○ Suburban (residential area on the outskirts of a city)

○ Rural (settled place outside of a city)

In which state do you currently live?

▼ Alabama ... Wyoming

This ladder represents where people stand in their communities. People define community in different ways; please define it in whatever way is most meaningful to you. The top of the ladder are people who are best off – those with the most money, high education, and the most respected jobs. At the bottom are people who are the worst off – those with the least money, minimal education, and the least respected jobs, or no job.

Where would you place yourself on this ladder? Please choose the number corresponding to the step where you think you currently stand, relative to other people in the community you live in.

▼ 1 ... 10

Have you ever owned a dog?

○ Yes

○ No

For how many cats are you currently the primary caregiver?

- ○ 1
- ○ 2
- ○ 3
- ○ 4
- ○ 5+

Have you owned a kitten or adult cat?

	Yes	No
Kitten (younger than 1 year old)	○	○
Adult cat (1 year or older)	○	○

Do you have any experience working with cats in a professional or volunteer setting (excluding cat ownership)?

- ○ Yes
- ○ No

In what capacity did or do you work with cats in a professional or volunteer setting? (Please select all that apply)

- ☐ Behaviorist
- ☐ Groomer
- ☐ Cat sitter
- ☐ Animal shelter
- ☐ Kennel staff/volunteer
- ☐ Trainer
- ☐ Working in a veterinary field (e.g. veterinarian, veterinary staff, veterinary student)
- ☐ Other (please specify)

____

How many years of combined experience do you have working with cats in a professional or volunteer setting?

- ○ Less than 1 Year
- ○ 1-5 years
- ○ 6-10 years
- ○ 11-15 years
- ○ 16+ years

How often do any of your cats display the following behavioural issues?

	Always	Often	Sometimes	Rarely	Never
Aggression (e.g. towards people and/or animals)	○	○	○	○	○
Excessive vocalisations	○	○	○	○	○
Destructive behaviours (e.g. scratching)	○	○	○	○	○
GI and ingestive disorders (e.g. eating non-food items, vomiting caused by eating too fast)	○	○	○	○	○
Excessive night time activity	○	○	○	○	○
Fears/phobias (e.g. noise phobias)	○	○	○	○	○
Separation Anxiety	○	○	○	○	○
Unwanted behaviours (e.g. jumping on counters, stealing food)	○	○	○	○	○
Abnormal behaviours (e.g. chasing tail, excessive licking)	○	○	○	○	○
Peeing or defaecating outside the litter box	○	○	○	○	○

The following questions pertain to socialisation programmes for kittens and/or adult cats (for example: Kitten Kindergarten)

Socialisation refers to the process of introducing animals of any age to new people, animals, and

environments (including new objects and noises). Often the goal of socialisation is to make the animal less reactive towards these new people, animals, and environments for the duration of their lives.

Socialisation programmes can include but are not limited to:
- training (example: best practice training methods, use of clickers for training, etc)
- exposure to new people, animals, and environments
- education about reading cat body language
- education about how to reduce common behavioral issues & unwanted behaviours

Have you heard of socialisation programs for kittens or adult cats before this survey?

	Yes	No	Unsure
For Kittens (younger than 1 year)	○	○	○
For Adult Cats (1 year or older)	○	○	○

Have you ever attended a socialisation class for any animal in your care in the past? (e.g. a puppy training or daycare programme)

- ○ Yes
- ○ No
- ○ Other ____

Where have you gotten information about cat or kitten socialisation from?
(Please select all that apply)

- ☐ Internet (websites, social media)
- ☐ Shelter (during the adoption process)
- ☐ Breeder
- ☐ Veterinarian
- ☐ Behaviorist
- ☐ Pet Store
- ☐ Books
- ☐ Groomer
- ☐ Other ____
- ☐ ⊗Have not gotten information about kitten/cat socialisation

Where have you gotten cats or kittens from in the past?
(please select all that apply)

- ☐ Shelter

- [ ] Breeder
- [ ] Pet Store
- [ ] Bred at home
- [ ] Gifted from friend/family
- [ ] Found/picked up from the street
- [ ] Other ____

How old was your cat(s) when you adopted them from a shelter?

- [ ] Kitten, 0 - 9 weeks
- [ ] Kitten, 10 weeks - less than 1 year
- [ ] Adult, 1 - 6 years old
- [ ] Adult, 7+ years old

How old was your cat(s) when you bought them from a breeder?

- [ ] Kitten, 0 - 9 weeks
- [ ] Kitten, 10 weeks - less than 1 year
- [ ] Adult, 1 - 6 years old
- [ ] Adult, 7+ years old

How old was your cat(s) when you bought/adopted them from a pet store?

- [ ] Kitten, 0-9 weeks

- ☐ Kitten, 10 weeks - less than 1 year
- ☐ Adult, 1 - 6 years
- ☐ Adult, 7+ years old

Has your veterinarian mentioned the following things about socialisation for your **kitten (younger than 1 year)**:

	Yes	No	Unsure
Training to respond to commands	○	○	○
Playtime with other **kittens**	○	○	○
Interaction with new people	○	○	○
Interaction with animals other than cats	○	○	○
Education about common behavioural issues (e.g. peeing outside the litter box)	○	○	○
Getting **kittens** comfortable with nail trims	○	○	○
Getting **kittens** used to handling	○	○	○
Leash/harness training	○	○	○
Carrier training	○	○	○
Other	○	○	○

Has your veterinarian mentioned the following things about socialisation for your **adult cat (1 year or older)**?

	Yes	No	Unsure
Training to respond to commands	○	○	○
Playtime with other **adult cats**	○	○	○
Interaction with new people	○	○	○
Interaction with animals other than cats	○	○	○
Education about common behavioral issues (e.g. peeing outside the litter-box)	○	○	○
Getting **adult cats** comfortable with nail trims	○	○	○
Getting **adult cats** used to handling	○	○	○
Leash/harness training	○	○	○
Carrier training	○	○	○
Other	○	○	○

Did the shelter mention the following things about socialisation for your **kitten (younger than 1 year)**?

	Yes	No	Unsure
Training to respond to commands	○	○	○
Playtime with other **kittens**	○	○	○
Interaction with new people	○	○	○
Interaction with animals other than cats	○	○	○
Education about common behavioural issues (e.g. peeing outside the litter box)	○	○	○
Getting **kittens** comfortable with nail trims	○	○	○
Getting **kittens** used to handling	○	○	○
Leash/harness training	○	○	○
Carrier training	○	○	○
Other	○	○	○

Did the shelter mention the following things about socialisation for your **adult cat (1 year or older)**:

	Yes	No	Not sure/Can't remember
Training to respond to commands	○	○	○
Playtime with other **adult cats**	○	○	○
Interaction with new people	○	○	○
Interaction with animals other than cats	○	○	○
Education about common behavioural issues (e.g. peeing outside the litter box)	○	○	○
Getting **adult cats** comfortable with nail trims	○	○	○
Getting **adult cats** used to handling	○	○	○
Leash/harness training	○	○	○
Carrier training	○	○	○
Other	○	○	○

Did your breeder mention the following things about socialisation for your **kitten (younger than 1 year)**?

	Yes	No	Unsure
Training to respond to commands	○	○	○
Playtime with other **kittens**	○	○	○
Interaction with new people	○	○	○
Interaction with animals other than cats	○	○	○
Education about common behavioural issues (e.g. peeing outside the litter box)	○	○	○
Getting **kittens** comfortable with nail trims	○	○	○
Getting **kittens** used to handling	○	○	○
Leash/harness training	○	○	○
Carrier training	○	○	○
Other	○	○	○

Did your breeder mention the following things about socialisation for your **adult cat (1 year or older)**:

	Yes	No	Not sure/Can't remember
Training to respond to commands	○	○	○
Playtime with other **adult cats**	○	○	○
Interaction with new people	○	○	○
Interaction with animals other than cats	○	○	○
Education about common behavioural issues (e.g. peeing outside the litter box)	○	○	○
Getting **adult cats** comfortable with nail trims	○	○	○
Getting **adult cats** used to handling	○	○	○
Leash/harness training	○	○	○
Carrier training	○	○	○
Other	○	○	○

Did the pet store mention the following things about socialisation for your **kitten (younger than 1 year)**?

	Yes	No	Unsure
Training to respond to commands	○	○	○
Playtime with other **kittens**	○	○	○
Interaction with new people	○	○	○
Interaction with animals other than cats	○	○	○
Education about common behavioral issues (e.g. peeing outside the litter box)	○	○	○
Getting **kittens** comfortable with nail trims	○	○	○
Getting **kittens** used to handling	○	○	○
Leash/harness training	○	○	○
Carrier training	○	○	○
Other	○	○	○

Did the pet store mention the following things about socialisation for your **adult cat (1 year or older)**?

	Yes	No	Not sure/Can't remember
Training to respond to commands	○	○	○
Playtime with other **adult cats**	○	○	○
Interaction with new people	○	○	○
Interaction with animals other than cats	○	○	○
Education about common behavioural issues (e.g. peeing outside the litter box)	○	○	○
Getting **adult cats** comfortable with nail trims	○	○	○
Getting **adult cats** used to handling	○	○	○
Leash/harness training	○	○	○
Carrier training	○	○	○
Other	○	○	○

Have you participated in any kitten (younger than 1 year old) socialisation programmes?

○ Yes

○ No

Please estimate the total number of hours it took to complete this kitten socialisation programme:

____

Was this class online or in-person

- ○ Online (pre-recorded classes, taken at your own pace)
- ○ Online (zoom call or in real time)
- ○ In-person
- ○ Both in-person and online

Where were the classes held?

- ○ Veterinary clinic
- ○ Shelter facility
- ○ Breeder's house/facility
- ○ Local university programme
- ○ Private behaviour consulting business
- ○ Other ____

Did the socialisation programme focus on any of the following:

	Yes	No	Not sure / can't remember
Lessons on cat body language/behaviour	○	○	○
Training cat to respond to commands	○	○	○
Playtime with other kittens	○	○	○
Kitten interactions with new people	○	○	○
Interactions with animals other than cats	○	○	○
Education about common behavioural issues (e.g. peeing outside the litter box)	○	○	○
Getting kittens comfortable with nail trims	○	○	○
Getting kittens used to handling	○	○	○
Leash/Harness training	○	○	○
Carrier training	○	○	○
Homework - At home tasks with your kitten	○	○	○
Other	○	○	○

If you were able to enroll your future adult cat or kitten in a socialisation programme, would you?

	Yes	No	Not sure
Kitten (younger than 1 year)	○	○	○
Adult Cat (1 year or older)	○	○	○

Please rate the importance of the following reasons for wanting to enroll your future **kitten (younger than 1 year)** in a socialisation programme

	Not at all Important	Slightly Important	Moderately important	Very Important	Extremely Important
Improve kitten's cooperation at the veterinary clinic	○	○	○	○	○
Improving kitten's health	○	○	○	○	○
Getting help for existing kitten behaviour problems	○	○	○	○	○
Reducing future cat behaviour problems	○	○	○	○	○
Want to be able to take kitten on leash	○	○	○	○	○
Want to learn how to train my kitten	○	○	○	○	○
Strengthen the bond with your kitten	○	○	○	○	○
Improving kitten's relationship with other cats in my home	○	○	○	○	○
Want cat to get along with other animals (non-cats)	○	○	○	○	○

Another reason (please explain)	○	○	○	○	○

Please rate the importance of the following reasons for wanting to enroll your future **adult cat (1 year or older)** in a socialisation programme

	Not at all Important	Slightly Important	Moderately important	Very Important	Extremely Important
Improve cat's cooperation at the veterinary clinic	○	○	○	○	○
Improving cat's health	○	○	○	○	○
Getting help for existing cat behaviour problems	○	○	○	○	○
Reducing future cat behaviour problems	○	○	○	○	○
Want to be able to take cat on leash	○	○	○	○	○
Want to learn how to train my cat	○	○	○	○	○
Strengthen the bond with your cat	○	○	○	○	○
Improving cat's relationship with other cats in my home	○	○	○	○	○
Want cat to get along with other animals (non-cats)	○	○	○	○	○

Another reason (please explain)	◯	◯	◯	◯	◯

Please rate the importance of the following reasons for **not** wanting to enroll your future **kitten (younger than 1 year)** in a socialisation programme

	Not at all Important	Slightly Important	Moderately Important	Very Important	Extremely Important
Price	○	○	○	○	○
Transportation challenges	○	○	○	○	○
Travel with kitten is too stressful	○	○	○	○	○
Challenges with placing kitten into carrier	○	○	○	○	○
No need for kitten to be exposed to new animals	○	○	○	○	○
No need for kitten to be exposed to new people	○	○	○	○	○
No need for kitten to be exposed to new environments	○	○	○	○	○
I don't have the time	○	○	○	○	○
Don't want to change kitten's behaviour	○	○	○	○	○
Concern over structure of class	○	○	○	○	○

Concern over the health risk of kitten's exposure to other cats	○	○	○	○	○
Other reason (please explain)	○	○	○	○	○

Please rate the importance of the following reasons for **not** wanting to enroll your future **adult cat (1 year or older)** in a socialisation programme

	Not at all Important	Slightly Important	Moderately Important	Very Important	Extremely Important
Price	○	○	○	○	○
Transportation challenges	○	○	○	○	○
Travel with cat is too stressful	○	○	○	○	○
Challenges with placing cat into carrier	○	○	○	○	○
No need for cat to be exposed to new animals	○	○	○	○	○
No need for cat to be exposed to new people	○	○	○	○	○
No need for cat to be exposed to new environments	○	○	○	○	○
I don't have the time	○	○	○	○	○
Don't want to change cat's behaviour	○	○	○	○	○
Concern over structure of class	○	○	○	○	○
Concern over the health risk of cat's exposure to other cats	○	○	○	○	○

Other reason (please explain)	○	○	○	○	○

How important do you think it is to include the following components into a **kitten (younger than 1 year)** socialisation programme:

	Extremely important	Very important	Moderately Important	Slightly important	Not at all important
Education about cat body language & behaviour	○	○	○	○	○
Learn basic training (i.e. training kitten to respond to commands)	○	○	○	○	○
Interaction with other kittens	○	○	○	○	○
Interaction with other animals (non-cats)	○	○	○	○	○
Interaction with new people	○	○	○	○	○
Expose kitten to new environments (including new objects & noises)	○	○	○	○	○
Learn about ways to reduce problem behaviours	○	○	○	○	○
Litter box training	○	○	○	○	○
Getting kitten used to handling	○	○	○	○	○

Getting kitten comfortable with nail trims	○	○	○	○	○
Carrier training for travel	○	○	○	○	○
Getting kitten to wear a harness & walk on leash	○	○	○	○	○
Homework - At home tasks with your kitten	○	○	○	○	○
Other	○	○	○	○	○

How important do you think it is to include the following components into a **adult cat (1 year or older)** socialisation programme:

	Extremely important	Very important	Moderately Important	Slightly important	Not at all important
Education about cat body language & behaviour	○	○	○	○	○
Learn basic training (i.e., training cat to respond to commands)	○	○	○	○	○
Interaction with other cats	○	○	○	○	○
Interaction with other animals (non-cats)	○	○	○	○	○
Interaction with new people	○	○	○	○	○
Expose cats to new environments (including new objects & noises)	○	○	○	○	○
Learn about ways to reduce problem behaviours	○	○	○	○	○
Litter box training	○	○	○	○	○
Getting cat used to handling	○	○	○	○	○

Getting cat comfortable with nail trims	○	○	○	○	○
Carrier training for travel	○	○	○	○	○
Getting cat to wear a harness & walk on leash	○	○	○	○	○
Homework - At home tasks with your cat	○	○	○	○	○
Other	○	○	○	○	○

How important do you think socialisation is for kittens/cats at the following ages:

	Extremely important	Very important	Moderately important	Slightly important	Not at all important
2 - 9 weeks of age	○	○	○	○	○
10 weeks - 1 year old	○	○	○	○	○
Over 1 year - 6 years old	○	○	○	○	○
7 - 10 years old	○	○	○	○	○
11+ years old	○	○	○	○	○

How much would you be willing to pay for the entire Kitten Kindergarten Socialisation Programme below:

- 1 hour in-person class once per week for three weeks
- Kittens would be introduced to other humans as well as other kittens

- You would be asked to complete short "homework" assignments with your kitten in between classes.

(The answer to this question should be a number value only, no other answers will be accepted)

____

Is there anything else you want to tell us about your thoughts on socialisation for cats and kittens?

____

____

____

____

____

Thank you for your time spent taking this survey! The purpose of this questionnaire is to better understand cat owner attitudes towards socialisation, and attempt to find out how likely cat owners are to want to participate in socialisation or kitten kindergarten classes with their cats.
